# Idiopathic Chondrolysis of the Hip in Two Adolescent Females: A Six-Year Follow-Up

**DOI:** 10.7759/cureus.39233

**Published:** 2023-05-19

**Authors:** Mohamad A Kawas, Syed Intekhab Alam, Faisal Hamad

**Affiliations:** 1 Orthopaedics, Hamad General Hospital, Doha, QAT; 2 Musculoskeletal Radiology, Hamad General Hospital, Doha, QAT

**Keywords:** pelvic tilt, limping gait, range of motion, hip joint, idiopathic chondrolysis

## Abstract

Idiopathic chondrolysis of the hip (ICH) is a rare condition with only a few cases reported in the literature. The average age at the onset of the disease is 11 years, with females having six times higher incidence than males. We report two cases of ICH in two medically free 10-year-old females who presented with atraumatic insidious hip pain and limping. No significant past medical, surgical, or family history was recorded. Laboratory studies were within normal limits, and the imaging showed the pathogenic changes of hip chondrolysis. Both cases were treated conservatively, and regular follow-ups in the clinic showed progressive limitation of the hip range of motion with arthritic changes on plain radiographs. Altogether, ICH is rare and can be misdiagnosed as inflammatory or infectious hip arthritis. Clinical assessment and image interpretation can lead to early diagnosis. Pain management and physical therapy with a prolonged period of protected weight-bearing are the mainstays of treatment.

## Introduction

Idiopathic chondrolysis of the hip (ICH) has a long history of reporting in the literature, with Waldenström being the first to report it [1]. Hip chondrolysis has a multifactorial etiology, including slipped capital femoral epiphysis, trauma, septic arthritis, prolonged periods of immobilization, or can be idiopathic (ICH) [2-7]. ICH is characterized by rapid and substantial damage to the articular cartilage in the absence of any known causes [8]. Outcomes may vary from complete recovery to almost fibrous ankyloses [9-11]. ICH affects females six times more commonly than males and is mostly unilateral [6,8,12]. The average age at the onset of the disease is 11 and ranges from 3-20 years of age with almost even distribution worldwide [3,4,7,10].

## Case presentation

Case one

**Figure 1:**
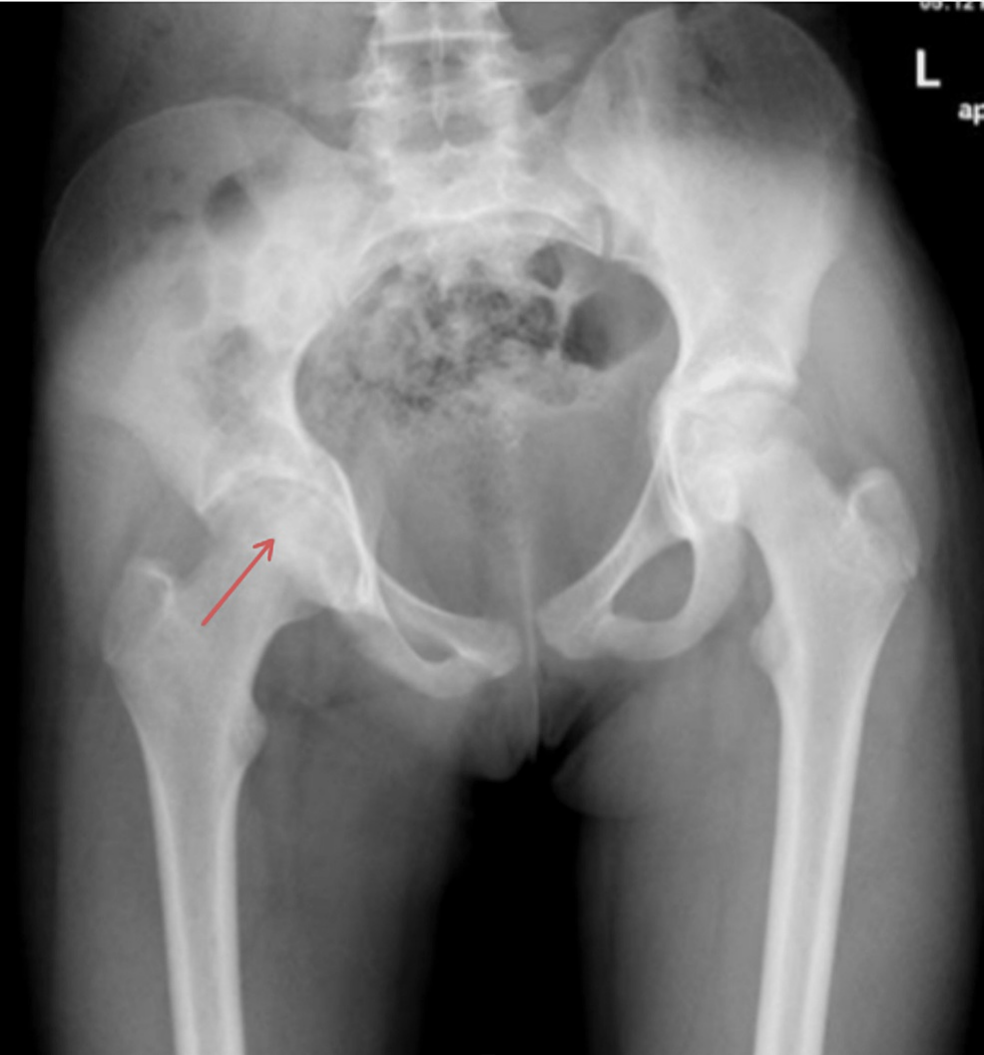
Anteroposterior pelvis radiograph showing mild medial joint space narrowing of the right hip with normal femoral head and pelvic tilt to the right side (red arrow).

**Figure 2:**
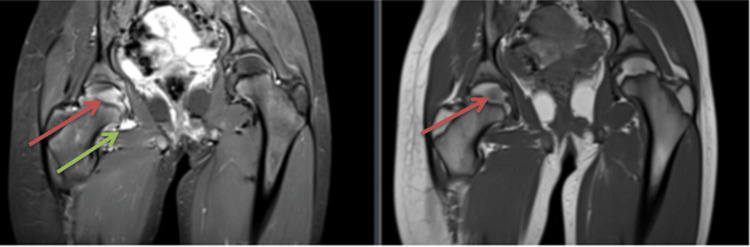
T2-weighted image with a fat-saturation sequence (left). T1-weighted image: coronal MRI hip (right) showing a band-like rectangular focal abnormal signal on the right femoral head, low on the T1-weighted image (red arrow on the right side) and bright on the T2-weighted image with a fat-saturation sequence (red arrow on the left side). Abnormal bright signal intensity is present on the T2-weighted image representing the right hip joint effusion (green arrow on the left).

**Figure 3:**
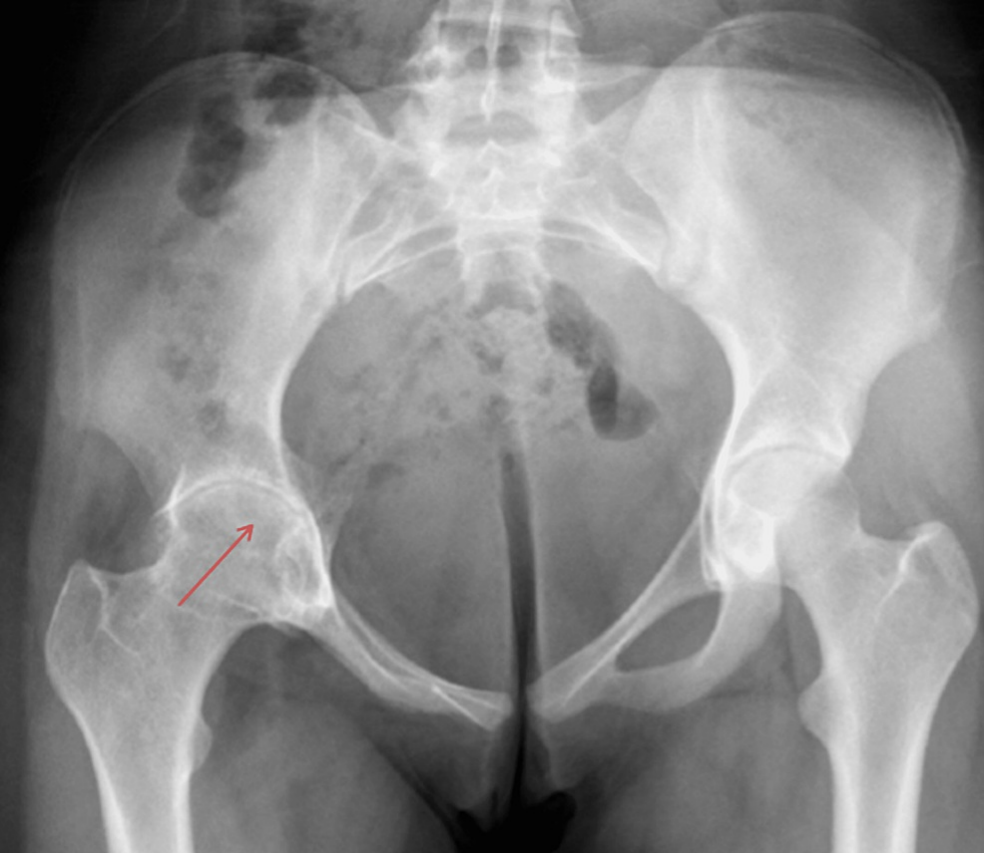
Follow-up pelvis anteroposterior radiograph showing concentric joint space narrowing on the right side with increased central edge angle with a mild pelvic tilt to the right side (red arrow).

A 10-year-old female presented with progressively worsening right hip pain of five months duration. There was no history of trauma, previous illness, or family history of rheumatologic diseases. She was not able to bear weight on the right side. On examination, she had an antalgic gait. There was no erythema, swelling, or warmth over the right hip, but she had a painful active and passive range of motion. Initially, there was a mild limitation in adduction and internal rotation of the hip with pain. Complete blood count, erythrocyte sedimentation rate, and C-reactive protein were normal. Initial radiographs of the pelvis revealed tilting of the pelvis toward the right side with slight asymmetry and reduction of the joint space on the right compared to the left, and normal size and shape of the femoral head with a smooth outline on both sides without apparent bone destruction or sclerosis (Figure 1). Magnetic resonance imaging (MRI) of the hip showed a band-like rectangular focal abnormal signal on the right femoral head, a pathognomonic MRI sign of idiopathic chondrolysis. It was low on the T1-weighted image and bright on the T2-weighted image with a fat-saturation sequence (Figure 2). She was initially treated with bed rest, skin traction, and non-steroidal anti-inflammatory drugs (NSAIDs) for two weeks, followed by physical therapy and non-weight-bearing ambulation with crutches. She was discharged home and regularly followed up over the next six years, which revealed a progressive worsening of her symptoms, resulting in painful limitation of movements of the right hip, which was associated with ipsilateral knee pain on prolonged walking. Her last clinic visit revealed stiffness in her right hip with only 20° of flexion, 10° of internal rotation, and 10° of adduction. Additionally, radiographs revealed concentric joint space narrowing (Figure 3).

Case two

**Figure 4:**
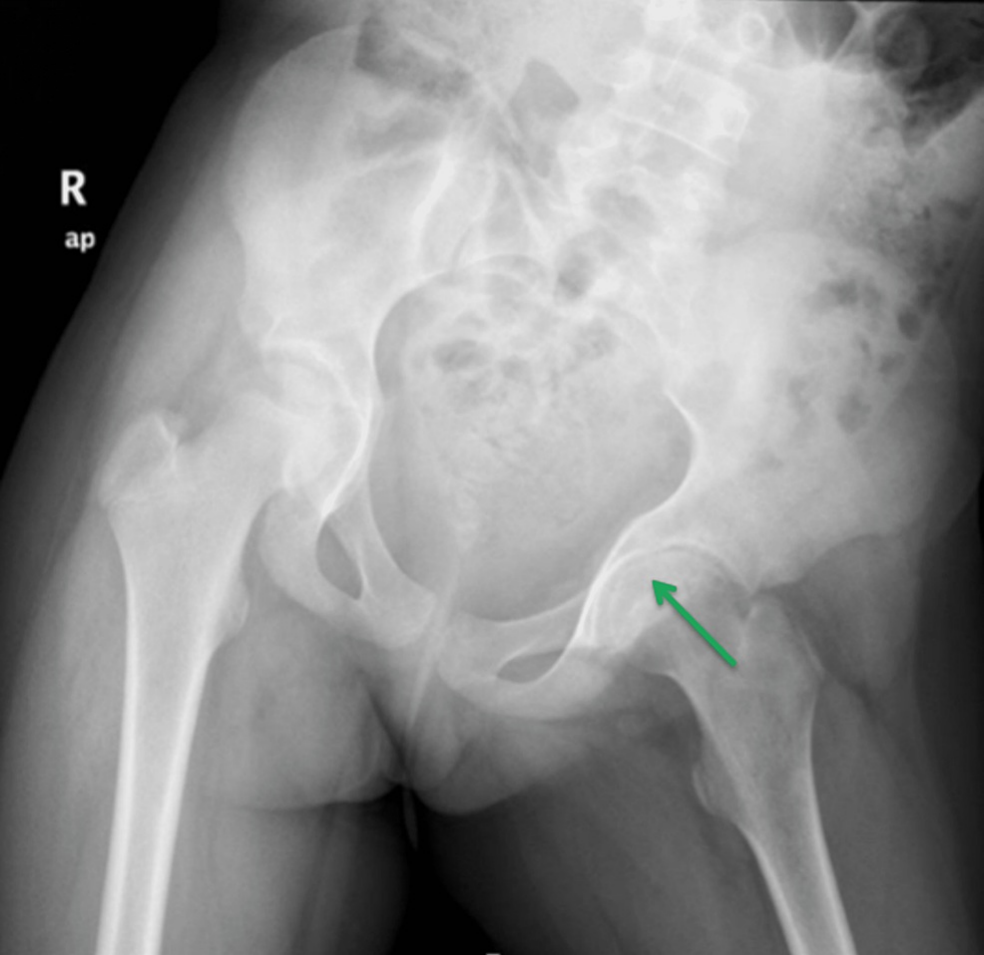
Initial anteroposterior pelvis radiograph showing concentric narrowing of the left hip joint space and reduced bone density (green arrow). Pelvic tilt to the left side can be seen.

**Figure 5:**
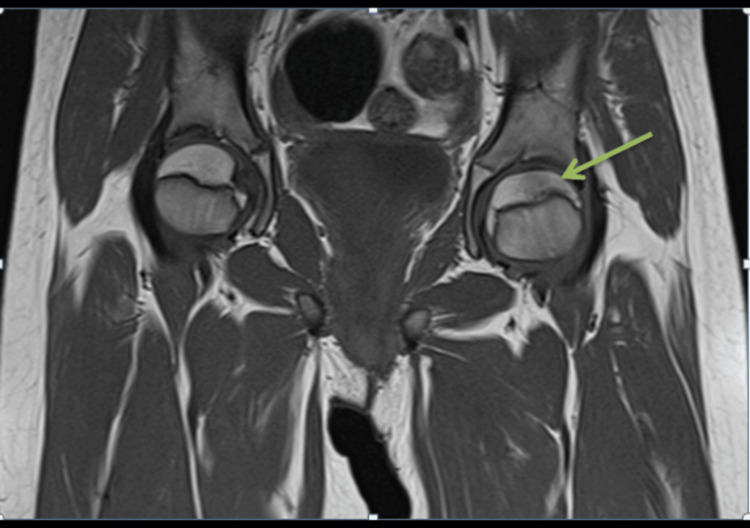
Coronal hip MRI showing a band-like rectangular focal abnormal signal on the left femoral head, which is low on the T1-weighted image (green arrow).

**Figure 6:**
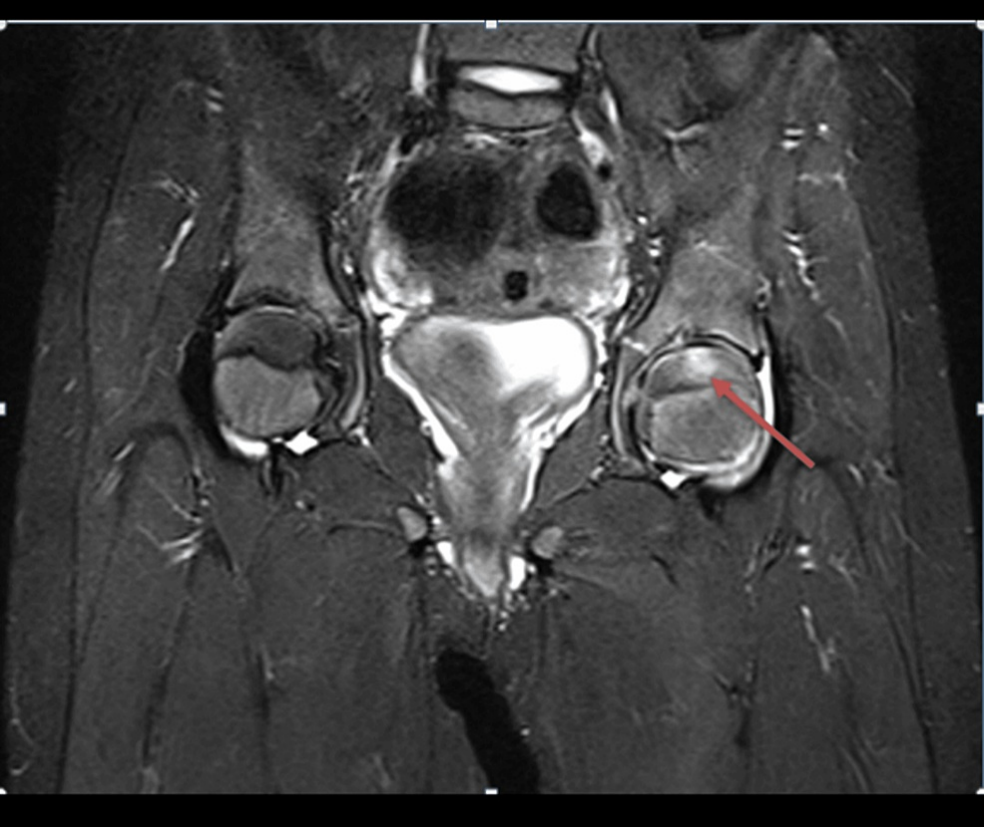
Coronal hip MRI (T2-weighted image with fat saturation) showing a band-like rectangular focal abnormally high signal on the left femoral head (red arrow).

**Figure 7:**
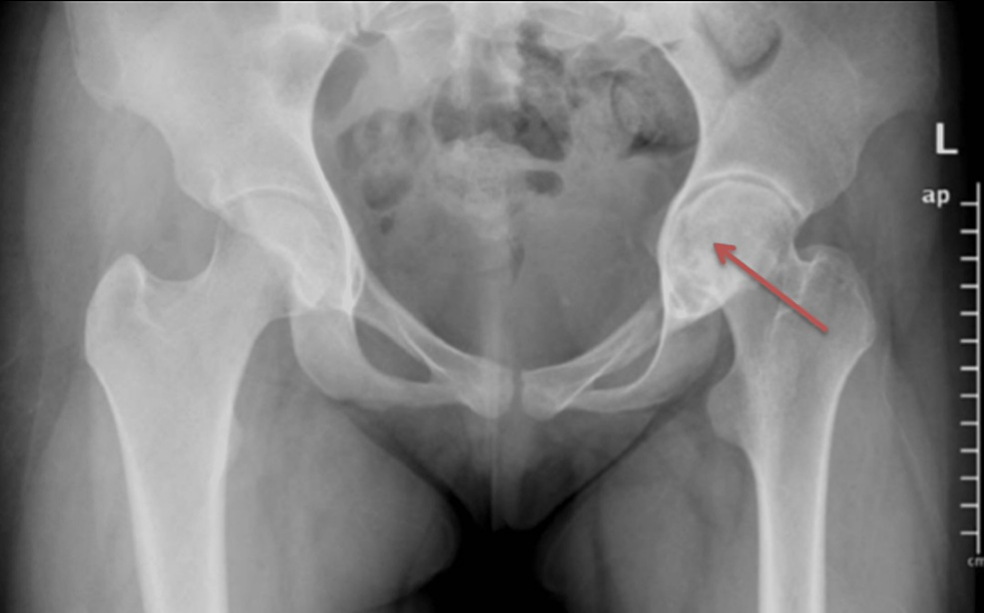
Follow-up pelvis anteroposterior radiograph showing concentric joint space narrowing with osteopenia (red arrow).

A 10-year-old girl presented with atraumatic left hip pain and limitation of movements with limping for two months. She had no personal or family history of any significant illnesses or rheumatologic conditions. On examination, she had a painful limitation of the left hip range of motion and an inability to bear weight. There was no erythema or swelling at the hip. Basic laboratory tests were normal, *Salmonella *and *Brucella *screening tests were negative, and rheumatoid factor and antinuclear antibody were within normal limits. A pelvic radiograph showed concentric narrowing of the left hip joint space and pelvic tilting (Figure 4). Hip MRI showed a band-like rectangular focal abnormal signal on the left femoral head, which was low on T1 (Figure 5) and bright on T2 sequences with fat saturation (Figure 6). Initially, the patient was admitted to the hospital and treated with analgesics, bed rest, and skin traction. She was discharged on NSAIDs and referred for physiotherapy and was regularly followed up in the orthopedics outpatient clinic for six years. However, the hip joint became progressively stiff. During the last clinical follow-up, she had limping gait with a limited left hip range of motion, especially internal rotation which was almost zero, and had adduction/abduction of 10° each, with an extension/flexion of 30° and 70° each. Her last follow-up radiographs showed concentric joint space narrowing with osteopenia (Figure 7).

## Discussion

We report two cases of ICH in adolescent females with no apparent underlying etiology or risk factors. Jones reported a series of nine cases of chondrolysis of the hip joint in black adolescent girls, seven of whom had no obvious underlying etiology [13]. In 1975, Duncan et al. encountered five cases of “bizarre stiff hip” and quoted them as ICH [14]. ICH is a disease of adolescents with a typical history of two to three months of insidious pain in the inguinal region, anterior thigh, or knee, with gradual restriction of hip range of motion [6,10,15,16]. Patients also present with a short-legged, antalgic gait and a flexed, abducted, externally rotated posture with no signs of inflammation or tenderness on palpation in a progressive manner [6,10]. Fixed hip deformities can lead to pelvic obliquity due to leg length discrepancy or an increase in compensatory lumbar lordosis [10]. Radiographic changes take several weeks to months to appear and may show narrowing of the affected hip joint space, peri-articular osteopenia, subchondral bone erosion, and/or ipsilateral pelvic tilt [8,12,15,17]. The space between the femur and acetabulum is normally 3.5-7 mm on plain radiographs. Reduction to less than 3 mm with osteopenia in the absence of osteophytes can be diagnostic of ICH [15]. Earlier it was thought that the disease always results in fibrous ankylosis of the hip joint [3]. However, it is now known that almost 50% of cases may have an acceptable clinical outcome in terms of function and range of motion with conservative treatment [6,15]. MRI can detect changes at an earlier stage compared to plain radiographs [15]. Initial MRI shows abnormal signals at the proximal femoral epiphysis with edema in the ipsilateral acetabulum, which may or may not be accompanied by joint fluid [12]. Historically, chondrolysis of the hip was treated surgically with soft tissue releases, osteotomies, hip arthrodesis, or arthroplasty. With the understanding of the natural history of the disease and the potential for good functional outcomes without surgery [11], orthopedic surgeons opted for conservative treatment, with a focus on pain relief, physiotherapy, and joint offloading. Among conservative options, NSAIDs combined with prolonged protected weight-bearing and aggressive physiotherapy have shown clinical improvement in more than half of acute cases [10].

## Conclusions

ICH is a rare entity and is often misdiagnosed as inflammatory or chronic infective arthritis. Adequate and timely clinical assessment and image interpretation can lead to early diagnosis. Various treatments are advocated such as comprehensive rehabilitation, immunosuppressive drugs such as etanercept, arthroscopic debridement, and circumferential capsulectomy with aggressive mobilization of the hip. Nevertheless, there is still a lack of consensus on effective treatment methodology to delay or treat the progress of disease which warrants more studies on this subject.
